# Effect of Sulindac Binary System on In Vitro and In Vivo Release Profiles: An Assessment of Polymer Type and Its Ratio

**DOI:** 10.1155/2016/3182358

**Published:** 2016-10-20

**Authors:** Gamal A. Shazly

**Affiliations:** ^1^Department of Pharmaceutics, King Saud University, P.O. Box 2457, Riyadh 11451, Saudi Arabia; ^2^Department of Industrial Pharmacy, Faculty of Pharmacy, Assiut University, Assiut 71526, Egypt

## Abstract

The bioavailability of sulindac (SDC), a nonsteroidal anti-inflammatory drug, is low due to poor aqueous solubility and poor dissolution rate. For this reason it is necessary to enhance the solubility and enhance dissolution of the drug by dispersing SDC in polyethylene glycols 6000 (PEG 6000) and polyvinyl pyrrolidone 40000 (PVP 40000) matrices using the coevaporation technique. Studying the influence of SDC to polymer ratio on drug content, percent yield, particle size, and in vitro release was performed. Differential scanning calorimetry, X-ray diffraction, and scanning electron microscopy were used to characterize any change in crystal habit of SDC in the prepared formulae. The anti-inflammatory effect of SDC was studied using the hind paw edema model. It was found that incorporation of SDC in PEG 6000 and PVP 40000 matrices resulted in improving the dissolution rate, which was found to depend on the polymer and its weight ratio of the drug. It is clearly obvious that the dissolution rate was remarkably improved in drug PVP 40000 molecular dispersions when compared to drug PEG 6000 systems. Solid dispersion of SDC in PEG and PVP improved the anti-inflammatory effect of SDC and it was found that formula SDV5 exhibited a more pronounced inhibition of swelling than other formulae.

## 1. Introduction

The proper dosage form of targeting and timely release of a drug is very important to be prepared to ensure the optimum therapeutic effect. Huge numbers of potential drugs are suffering from poor water solubility and/or low dissolution rate. Amidon et al. [1] have classified these drugs in the Biopharmaceutical Classification System (BCS) as class II drugs. The improvement of drug solubility is necessary for improving drug dissolution and subsequently drug bioavailability. Various techniques such as mechanization, nanoparticle formation, modifications of the crystal habit, polymorphism, salt formation, solubility, complexation, and drug dispersion in carriers are used for improving the solubility and the bioavailability of poorly water soluble drugs [2]. One of the techniques used for enhancing the solubility and dissolution of poorly water soluble drugs and hence improving their bioavailability is molecular dispersions of drugs in water soluble carriers [3]. In molecular dispersion, one or more active therapeutic components are dispersed in an inert carrier matrix [4]. Moreover, in molecular dispersion, (1) drug particle sizes are reduced, resulting in an increase in the surface area, (2) the crystalline form of the drug is changed into an amorphous form, (3) higher particle porosity is produced, and these finally result in enhancing drug dissolution rate and hence bioavailability [5, 6], breaking down the crystal lattice [7], or increasing the drug wettability by surrounding drug molecules by hydrophilic carriers [5]. Formulation of molecular dispersion has the advantages of being easy to be performed and of being applicable to many types of drugs [8]. In contrast, other approaches have disadvantages such that salt formation is limited to acidic or basic drugs and micronization techniques are sometimes insufficient to enhance drug solubility and drug release in the gastrointestinal tract due to the imperfect size reduction.

Sulindac (SDC) is a nonsteroidal anti-inflammatory drug related to BCS class II, whose absorption is dissolution rate limited. SDC is a class II drug, according to the Biopharmaceutics Classification System because it has high membrane permeability and low water solubility [9]. Similar to other NSAIDs, SDC is used for management of acute inflammatory and chronic situations. It is a prodrug that is altered in the body to the active form. More precisely, the drug is transformed to a sulfide by liver enzymes, is excreted in the bile, and then is reabsorbed from the intestine. Thus, constant blood levels of the drug are maintained with reduced gastrointestinal side effects. It was reported by some studies that SDC is relatively less irritating to the stomach than other NSAIDs drugs except COX-2 inhibitor class. Its exact mechanism as anti-inflammatory is unidentified, but it is believed to act on COX-1 and COX-2 enzymes, inhibiting the prostaglandin synthesis. SDC seems to have a property, independent of COX inhibition, of reducing the growth of polyps and precancerous lesions in the colon, especially in association with familial adenomatous polyposis, and may have other anticancer properties [10, 11]. SDC is an effective tocolytic agent and may be used in the treatment of preterm labor. In common with other NSAIDs, sulindac is currently being investigated for the role in the management of Alzheimer's disease. Since this drug was found such that the sulfoxide functional group can be reduced by methionine sulfoxide reductase A (MsrA), a possible antioxidative capability is being discussed.

The objective of this work is to enhance the solubility, the rate of dissolution and hence may improve the bioavailability of SDC by molecular dispersion with PEG and PVP. The physicochemical properties, in vitro release, and in vivo characteristics of SDC in these dispersions are studied.

## 2. Materials and Methods

### 2.1. Materials

Sulindac (SDC) and polyvinyl pyrrolidones 40000 (PVP 40000) were purchased from Sigma Chemical Co., USA. Polyethylene glycol 6000 (PEG 6000) and dichloromethane were purchased from Fluka Chemica, Buch, Switzerland. Potassium dihydrogen orthophosphate and disodium hydrogen orthophosphate were purchased from El-Nasr Pharmaceutical Co., Egypt. Other materials and solvents are of reagent or analytical grade, and they were used without further purification.

### 2.2. Preparation of SDC Binary Systems

Binary coprecipitates of SDC with PEG 6000 or PVP 40000 in the weight ratio of 1 : 1, 1 : 2, 1 : 3, and 1 : 5 were prepared by coevaporation technique. Briefly, the weighed amounts of SDC and the polymers were dissolved in adequate amount of dichloromethane (Table 1). The mixture was stirred at room temperature (25°C) for 20 minutes, and then the solvent was removed under vacuum in a rotary evaporator, at a temperature of 40°C. Solid residue was dried in a desiccator for 24 h at room temperature (25°C), pulverized, and sieved (Sieve shaker, Rx-86-1, Cole-Parmer Instrument Co., USA). Powder samples less than 40 mesh were kept in closed containers away from the light and humidity pending the investigations [12].

### 2.3. Preparation of SDC-Polymer Physical Mixtures

In a glass mortar, physical mixtures having the same ratios of SDC and polymer were gently mixed and the mixtures were then passed through number 40 sieves.

### 2.4. Characterization of the SDC Binary Systems

#### 2.4.1. Determination of Percent SDC Content

An exactly weighed amount of solid dispersion and physical mixtures equivalent to 5 mg of SDC dissolved in 100 mL of methanol in stoppered conical flasks. The closed flasks were agitated on a rotary shaker for 1 hour. The resulting solution was then filtered through the filter paper. One mL of solution was diluted 10 times with 0.2 M phosphate buffer solutions and the absorbance was assayed by a UV-VIS for spectrophotometric assay at 327 nm (Shimadzu Corporation, Japan) to calculate the drug content using the following expression [13]:

(1) Percent  drug  content=practical  drug  content  in  solid  dispersionstheoretical  drug  content  in  solid  dispersions×100.

#### 2.4.2. Determination of SDC Percent Yield

The percent yield of SDC in solid dispersions and physical mixture can be determined by using the following expression:

(2) $$\begin{array}{c} Percent\;\;yield=\left(\frac{weight\;\;of\;\;solid\;\;dispersion}{weight\;\;of\;\;drug}+carriers\right)\times 100. \end{array}$$

#### 2.4.3. Differential Scanning Calorimetry (DSC)

DSC thermograms were performed for SDC binary systems compared to that of the individual components and physical mixtures in order to determine the extent of crystallinity of the drug in the presence of the studied polymers. Three to five mg of the samples were packed in aluminum pans and heated at a constant rate of 10°C/min, up to 300°C. The thermograms of the samples were achieved using differential scanning calorimeter (DSC-60, Shimadzu, Japan). The thermal analysis data were recorded using a TA 50I PC system with Shimadzu software programs. Indium standard was used to calibrate the DSC temperature and enthalpy scale. Nitrogen was used as purging gas at a rate of 30 mL/min.

#### 2.4.4. Powder X-Ray Diffraction (PXRD)

The X-ray diffractograms were achieved by means of Jeol XR diffractometer (Jeol, Tokyo, Japan). The radiation source was a copper (*ƛ* = 1.54184 Å) high-intensity X-ray tube operated at 35 kV and a current of 15 mA. The diffraction patterns were achieved using continuous scan mode with 2*θ* values ranging from 4 to 100 at a rate of 4 degrees/minute.

#### 2.4.5. Fourier Transform Infrared Spectroscopy (FTIR)

Small amount of each sample was mixed with potassium bromide. The mixture was then compressed into discs using hydraulic press. The disc is then scanned over a frequency range of 4000–500 cm^−1^ using FTIR spectrophotometer (FTIR-8400S, Shimadzu). IR Solution software (version 1.10, Marlborough, MA, USA) was utilized for analysis of the obtained IR data.

#### 2.4.6. Scanning Electron Microscopy (SEM)

The morphology of SDC, physical mixture, and solid dispersions with PEG 6000 and PVP 40000 were examined using scanning electron microscope (SEM) (Carl Zeiss EVO LS10; Cambridge, United Kingdom). Samples were put on stubs using both-side adhesive carbon tape (SPI Supplies, West Chester, USA) and covered with gold under vacuum using a Q150R sputter coater unit from Quorum Technologies Ltd. (East Sussex, United Kingdom) in an argon atmosphere at 20 mA for 120 seconds.

### 2.5. In Vitro Dissolution Studies

The in vitro dissolution of SDC from the binary systems and physical mixture was accomplished using USP dissolution apparatus II (Caleva Ltd., Model 85T), at 100 rpm using a continuous automated monitoring system. This system is composed of an IBM computer PK8620 series and PU8605/60 dissolution test software, PhilipsVIS/UV/NIR single beam eight-cell spectrophotometer Model PU 8620, Epson FX 850 printer, and Watson-Marlow peristaltic pump using in each flask a 900 mL phosphate buffer, pH 6.8, and the temperature was maintained at 37 ± 0.5°C. Twenty-five milligrams of SDC or equivalent amount of the binary and physical mixture was spread over the dissolution medium. At predetermined time intervals, absorbance was recorded automatically at 327 nm and the percentage of SDC dissolved was calculated as a function of time in triplicate and the mean was considered.

### 2.6. Anti-Inflammatory Study

In vivo anti-inflammatory activity was estimated on the basis of the inhibition of the volume of the hind paw edema made by injecting an irritant (formalin 1% w/v in 0.9% w/v saline) into the rat's paw [14].

#### 2.6.1. Selection of Animals

Adult male Wistar Albino rats aging approximately 3 months and weighting 150 ± 10 g were obtained from the Animal Care Center, College of Pharmacy, King Saud University, Riyadh, Saudi Arabia. The rats were retained in metabolic cages under controlled environmental conditions (25°C and a 12 h light/dark cycle). Animals had free access to pulverized standard rat pellet food and tap water. The protocol of this work has followed the instruction of the Research Ethics Committee of College of Pharmacy, King Saud University, Riyadh, Saudi Arabia. The animals were divided into five groups, each consisting of four rats. The first group was considered as control without taking any medicament. The other groups 2, 3, 4, and 5 were given 1 mL of a suspension SDC in methyl cellulose, methyl cellulose suspension, solid dispersion of SDC in PVP suspended in methyl cellulose, and solid dispersion of SDC in PEG suspended in methyl cellulose, respectively, at a dose of 2 mg/kg [15]. After half an hour, the animals were generally anesthetized by intraperitoneal injection of 1 mL of urethane (25% w/v). After one hour, 0.1 mL formalin (10% v/v) was injected subcutaneously into the plantar region of the right hind paw for all groups. At time intervals 1.5, 2.5, 3.5, and 4.5 hours, the inflammation was measured using 37140-plethysmometer (Ugo Basile SRL, Comerio, VA, Italy). The anti-inflammatory response (%) was calculated according to the following equation:

(3) $$\begin{array}{c} Response\%=\frac{\left(C-T\right)}{C}*100, \end{array}$$

where *C* is inflammation of right paw and inflammation of left paw for control rat and *T* is inflammation of right paw and inflammation of left paw for treated rat.

## 3. Results and Discussion

Various SDC molecular dispersions were formulated using PEG 6000 and PVP 40000 individually as carriers by coevaporation technique to improve the solubility as well as the dissolution of poorly aqueous soluble drug SDC.

The percent yield of the prepared SDC solid dispersions and physical mixtures was found within the range of 90.44 ± 3.14% to 97.38 ± 2.67% (Table 1). On the other hand, the percentage of drug content ranged from 98.11 ± 3.87% to 99.71 ± 1.64%, as described in Table 1. This indicates that SDC was homogeneously distributed in all these prepared solid dispersions and physical mixtures. These results are in accordance with those that have been found by Aejaz et al. [16].

**Figure 1:**
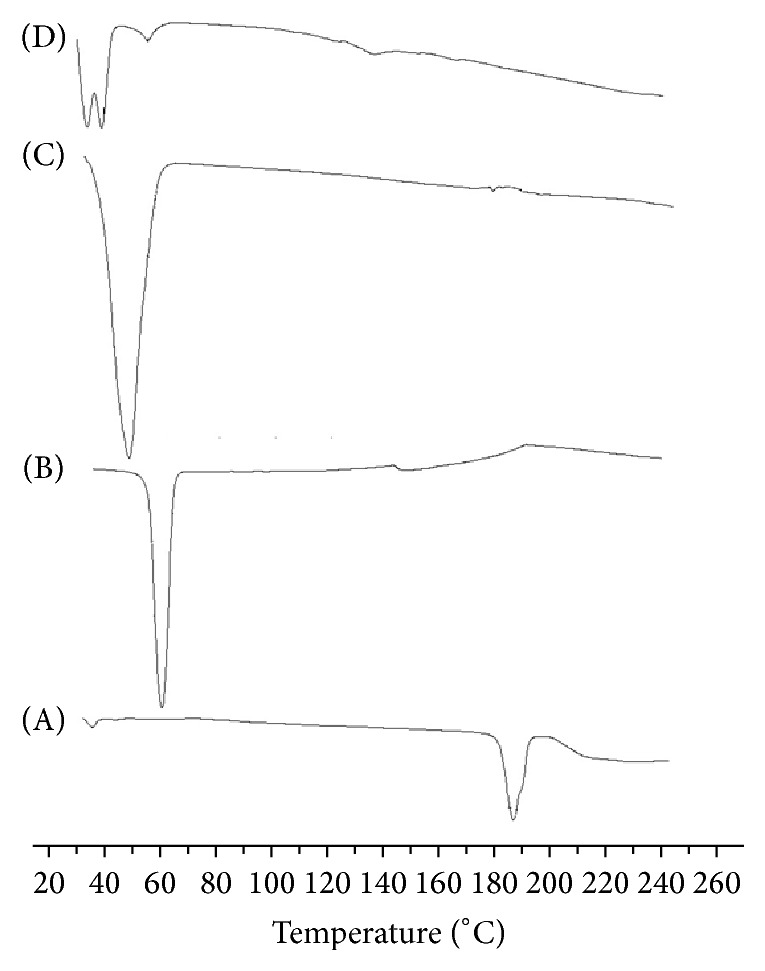
DSC data of (A) unprocessed SDC, (B) PEG 6000 alone, (C) SDE5, and (D) PME5.

**Figure 2:**
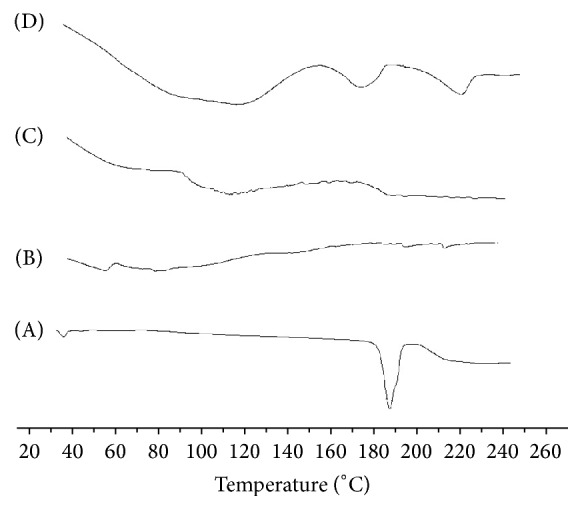
DSC data of (A) unprocessed SDC, (B) PVP 40000 alone, (C) SDV5, and (D) PMV5.

Figures 1 and 2 exhibit DSC thermograms of SDC alone, solid dispersions, and physical mixtures of SDC in both PEG 6000 and PVP 40000 in a ratio of 1 : 5 (drug : polymer). DSC of SDC displays an endothermic peak at 181.97°C, with enthalpy of infusion being Δ*H*_*f*_ = −59.70 J/g, which is equivalent to its melting point, at the rate of heating used (Figures 1 and 2). DSC thermograms of PEG 6000 displayed an endothermic peak around 60.43°C representing the melting point of the polymer (Figure 1). The shift of thermal features of SDC to lower melting point with very low intensity shows that some interaction between SDC and PEG might have happened. These results might be due to the formation of an amorphous solid solution. In case of physical mixture, the melting point also was broad and was moved to a lower melting peak. On melting, PEG has the property of being able to dissolve drugs that were complexed earlier, attaining their own melting point [17]. This occurrence may elucidate the shifting to a lower melting peak for SDC in the solid dispersions and physical mixture with PEG 6000, suggesting that SDC was completely dissolved in the liquid phase of PEG 6000. Similar findings have been reported by other authors [18, 19]. PVP is an amorphous polymer and its DSC thermograph in solid dispersions displays a broad endothermic effect ranging from 30.26°C to 123.85°C, with a peak at 65.68°C (Figure 2). The disappearance of the characteristic SDC peak might be due to the conversion to amorphous characteristics in the solid dispersions of SDC with PVP [20]. In case of physical mixtures, the endotherm peak was shifted somewhat to lower melting point. This may be due to solvent effects of molten polymer [21].

**Figure 3:**
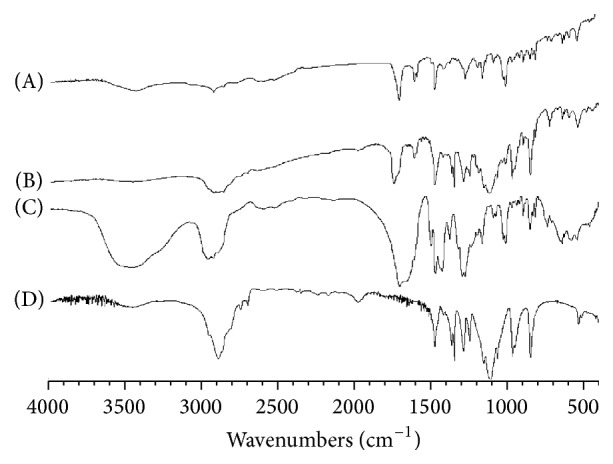
FTIR patterns for the untreated (A) drug (SDC) alone, (B) PME5, (C) SDE5, and (D) PEG 6000 alone.

**Figure 4:**
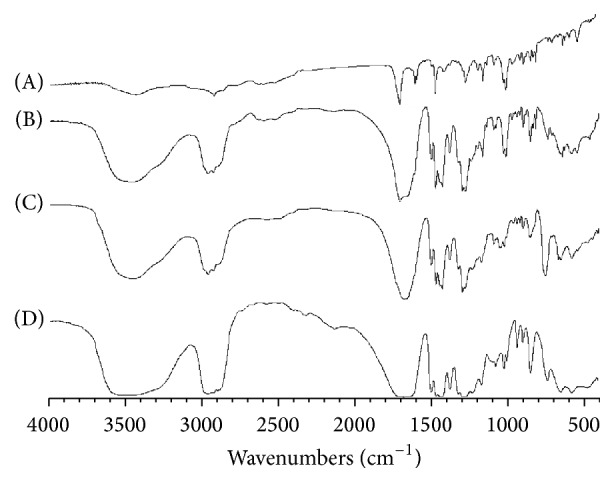
FTIR patterns for the untreated (A) drug (SDC) alone, (B) PMV5, (C) SDV5, and (D) PEG 6000 alone.

For further revealing of the interaction possibility between SDC and polymers in the solid state, FTIR spectroscopy was used. FTIR spectra of SDC, physical mixtures, and solid dispersions are represented in Figures 3 and 4. The FTIR spectrum of pure SCD exhibited characteristic peaks at 965.21 cm^−1^ (O–H bending out of plane), 3426.05 cm^−1^(O–H stretching), 1701.81 cm^−1^ (–C=O stretching), 1589.27 cm^−1^, 1602.8 cm^−1^ (–C=C stretching of aromatic), and some prominent bands such as 1415.61–1469.53 cm^−1^ (–C–O stretching). The FTIR spectra for SDC solid dispersions in both PEG 6000 and PVP 40000 matrices show absence of the characteristic peak of the pure drug at 3426.05 with the other characteristic peaks of the drug being broad and decreased in intensities, which might be due to drug interaction with the polymers, suggestive of the formation of an amorphous state of the drug.

**Figure 5:**
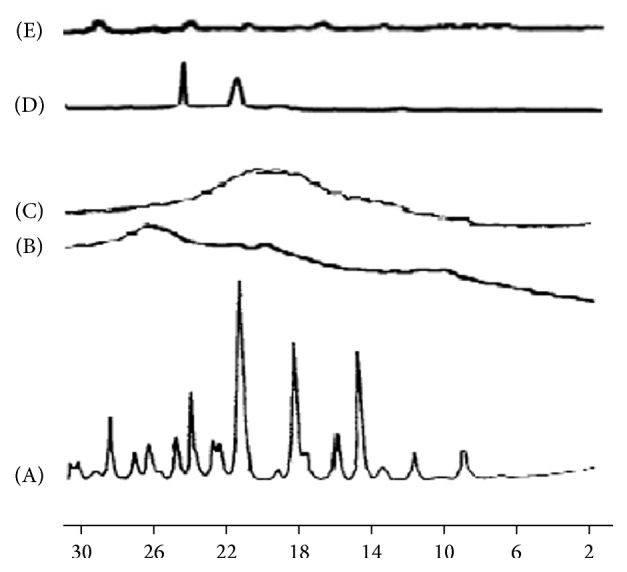
PXRD patterns for the untreated (A) drug (SDC) alone, (B) PVP 40000 alone, (C) SDV5, (D) PEG 6000 alone, and (E) SDE5.

The powder XRD patterns of PEG 6000, PVP 40000, and SDC binary (1 : 5) solid dispersions with PVP 40000 and PEG 6000 are shown in Figure 5. Intact SDC exhibits numerous diffraction peaks indicating the presence of the crystalline nature of SDC. The powdered PVP 40000 was amorphous where it shows only few peaks. The diffractogram of SDC-PVP (1 : 5) solid dispersion is more similar to that of PVP 40000 indicating the absence of the drug crystalline peaks. The powder XRD patterns of SDC-PEG solid dispersion systems at a drug : polymer ratio 1 : 5 reveal that no sharp peaks attributable to SDC are observed in the solid dispersion indicating that SDC crystals were transformed to a noncrystalline form during the cosolvent process. The PXRD results are well correlated with the DSC data.

**Figure 6:**
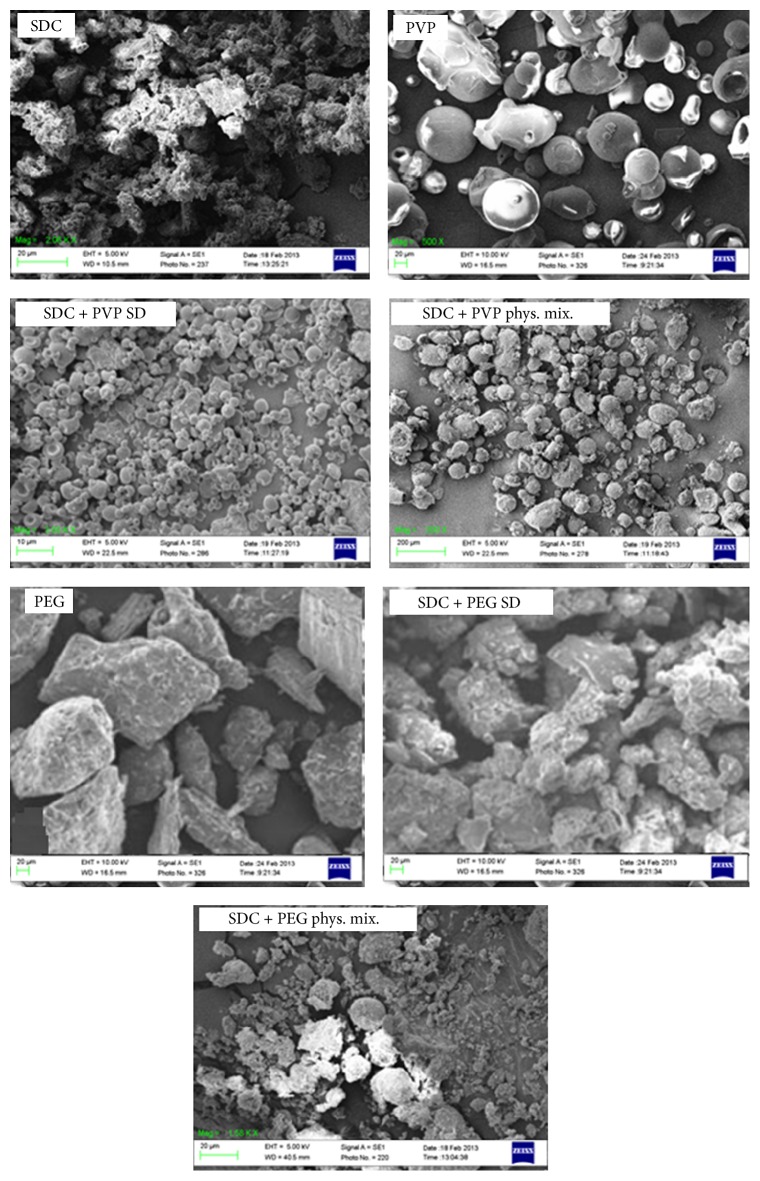
Scanning electron micrography of unprocessed SDC, PEG 6000 alone, PVP 40000 alone, SDV, SDE5, PMV5, and PME5.

The scanning electron micrograph images of SDC solid dispersions in PVP 40000 and PEG 6000 matrices at a drug : polymer ratio of 1 : 5 are displayed in Figure 6. SDC powder shows rough, irregular crystalline shapes, while PVP powder showed smooth spherical crystals ranging from 40 to 60 *µ*m. In case of SDC-PVP solid dispersion, the SEM images showed a homogeneous dispersion of the drug crystals in the polymer matrix with complete disappearance of the drug crystalline state. In contrast, SEM image of SDC-PVP physical mixture shows that drug crystals are present and embedded in the polymer matrix. In case of SDC-PEG 1 : 5 systems, SEM reveals that the drug exhibits amorphous nature in the polymer matrix in both the PEG solid dispersion and physical mixture.

**Figure 7:**
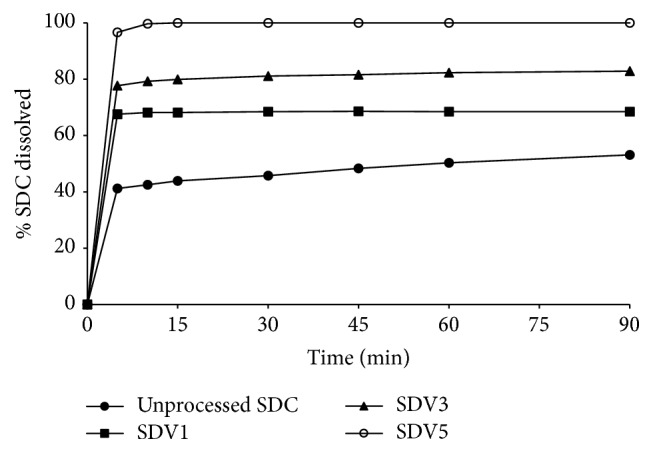
Dissolution study of unprocessed SDC and SDC-PVP solid dispersion systems at different polymer ratios in pH 6.8 phosphate buffer.

Figure 7 illustrates the dissolution pattern of SDC from PVP at different drug : polymer ratios, compared to the pure drug. The drug alone displays a slow dissolution rate, in which a burst dissolution of 41% was noted after 5 min followed by a slow released amount, where only 51% of the drug was dissolved within 90 min. Incorporation of SDC in PVP 40000 matrices resulted in improving the dissolution rate, and the improvement of the drug dissolution rate was found to depend on the polymer weight ratio. For example, 68% and 79% were dissolved after 10 min from SDC-PVP solid dispersions at SDC : PVP 40000 ratios of 1 : 1 and 1 : 3, respectively, while complete drug dissolution was obtained from SDC-PVP 1 : 5 solid dispersion at the same time.

**Figure 8:**
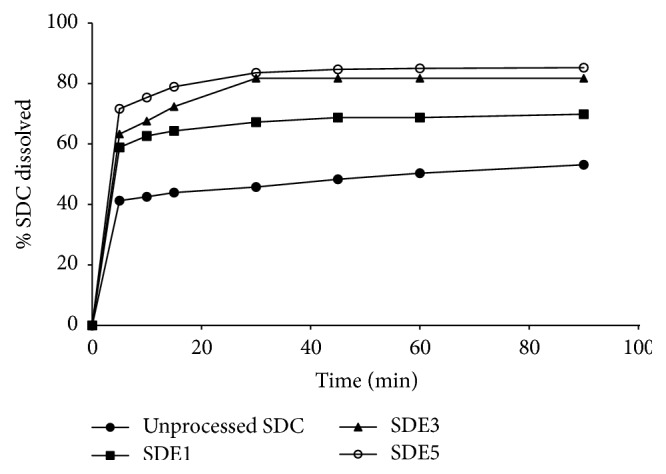
Dissolution study of unprocessed SDC and SDC-PEG solid dispersion systems at different polymer ratios in pH 6.8 phosphate buffer.

Figure 8 explains the in vitro dissolution profiles of SDC from the corresponding physical mixtures in PVP matrices. A small increase in the dissolution rate of the drug was exhibited in PVP physical mixtures, while the effect of polymer weight ratio on the dissolution rate was insignificant.

**Figure 9:**
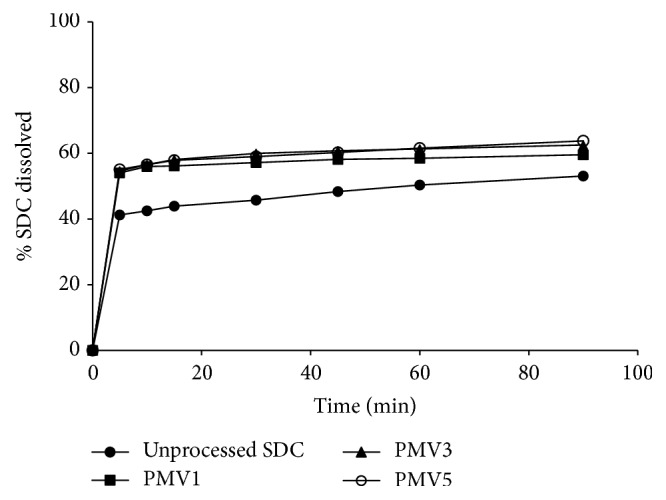
Dissolution study of unprocessed SDC and SDC-PVP physical mixtures at different polymer ratios in pH 6.8 phosphate buffer.

**Figure 10:**
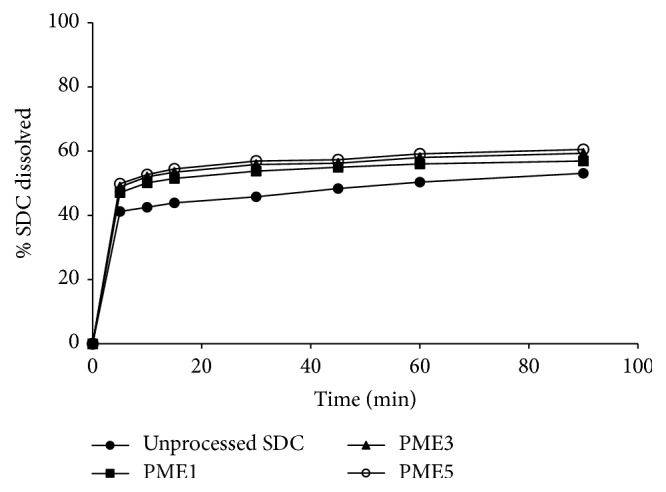
Dissolution study of unprocessed SDC and SDC-PEG physical mixtures at different polymer ratios in pH 6.8 phosphate buffer.

With regard to SDC-PEG 600 solid dispersion systems, the drug shows also enhanced dissolution rate as the weight ratio of the polymer was increased. After 10 minutes, 58.83%, 67.52%, and 75% were dissolved from SDC-PEG solid dispersions at SDC : PEG 6000 ratios of 1 : 1, 1 : 3, and 1 : 5, respectively, as shown in Figure 9. Moreover, very slight enhancement of the drug dissolution rate was observed in case of the corresponding physical mixtures of SDC in PEG matrices, as shown in Figure 10.

It is clearly evident that the drug dissolution rate was remarkably improved in PVP 40000 solid dispersions when compared to its PEG 6000 systems. According to Chiou and Riegelman [4], several factors could contribute to the enhanced drug dissolution performance of drug-polymer dispersed mixtures. Particle size reduction of the drug, improved wettability, and loss of crystallinity occurring during the coprecipitation are considered the principal factors responsible for the enhanced dissolution behavior of the drug. Furthermore, according to van den Mooter et al. [18], PVP was found to be effective in the prevention of such crystallization on the condition that the drug was formulated in molecular dispersions since physical mixing with the polymer led to crystallization. They have decided that the mechanism of the protective effect of PVP in the case of amorphous ketoconazole is not the drug-polymer interactions but mainly could be due to the polymer antiplasticizing effect, thus decreasing the diffusion of drug molecules and increasing the viscosity of the binary system necessary to form a lattice.

When the proportion of PVP in the solid dispersion is increased, the SDC dissolution rate also increases. This could be due to the fact that the solid dispersion was a homogeneous dispersion of the drug in the polymer matrix which is produced [22]. In addition, interaction forces (Van der Waals forces) between drug molecules are decreased in these mixed crystals, and as a result the dissolution of the drug from this solid dispersion is faster than from the pure drug. Moreover, the improved SDC dissolution rate might be due to the prevention of crystallization of the drug caused by PVP [2] as indicated from the DSC and PXRD data.

**Figure 11:**
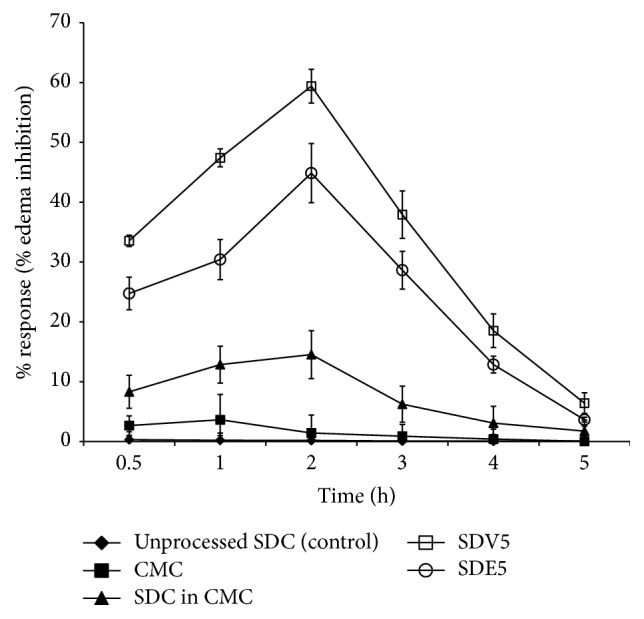
The anti-inflammatory activity of orally delivered formulae SDV5, SDE5, CMC, SDC in CMC, and unprocessed SDC (control). Data expressed as mean ± SD (*n* = 3). ^*∗*^Significant difference (*p* > 0.05).

The anti-inflammatory effect of SDC in the presence and the absence of polymers was performed using the paw edema method. Formulae SDV5 and SDE5 were chosen for this study. Each sample was suspended in carboxymethyl cellulose before it was given to the rats.  Figure 11 reveals the percent of the edema inhibition versus time for selected SDC formulae compared to SDC suspended in carboxymethyl cellulose, carboxymethyl cellulose suspension, and unprocessed SDC (control). It is noticeable that the presence of polymers significantly reduced the inflammation. The extent of edema reduction by SDC formulae was due to the effect of polymers on the dissolution and hence bioavailability of SDC. The anti-inflammatory effect of SDV5 formula exhibits rapid onset of action (highest edema inhibition) as compared to SDE5 formula. The extent of inhibition was more than 55% at 2 hours after formalin injection. This significant enhancement in the anti-inflammatory effect of SDC dispersed in the PVP polymer could be attributed to the improvement of SDC bioavailability as revealed from dissolution data. This was in accordance with that found by Jafar et al. [23] and Barzegar-Jalali et al. [24].

## 4. Conclusion

Molecular dispersion of the dug in PEG and PVP prepared by coevaporation method exhibited improvement of drug release and this is due to wettability and hydrophilic nature of these polymers. It may be concluded that the amount of the drug released depends on drug to the polymer ratio. Solid dispersion in PVP polymer showed the highest in vitro dissolution as compared to that in PEG polymer. SDV5 formula revealed an enhancement of in vivo anti-inflammatory activity as compared to pure drug and control. Finally, it could be obvious that enhanced drug dissolution and anti-inflammatory effect could be accomplished by formulating SDC as solid dispersion systems with PVP and PEG.

## Figures and Tables

**Table 1:** Yield and drug content of SDC solid dispersions and physical mixtures at different ratios.

Binary mixture	Formula number	Ratio	Yield (%)^*∗*^	Drug content (%)^*∗*^
SDC : PVP 40000 Solid dispersion	SDV1	1 : 1	94.24 ± 2.64	99.71 ± 1.64
	SDV3	1 : 3	95.37 ± 4.11	99.84 ± 2.71
	SDV5	1 : 5	97.38 ± 2.67	99.91 ± 3.18

SDC : PEG 6000 Solid dispersion	SDE1	1 : 1	92.31 ± 3.71	98.81 ± 4.11
	SDE3	1 : 3	93.11 ± 2.82	99.59 ± 3.54
	SDE5	1 : 5	95.27 ± 4.07	99.78 ± 2.45

SDC : PVP 40000 Physical mixture	PMV1	1 : 1	91.11 ± 5.24	98.44 ± 3.18
	PMV3	1 : 3	92.87 ± 3.48	99.67 ± 4.37
	SDV5	1 : 5	94.87 ± 2.97	99.17 ± 2.87

SDC : PEG 6000 Physical mixture	PME1	1 : 1	90.44 ± 3.14	98.11 ± 3.87
	PME3	1 : 3	93.18 ± 4.01	98.64 ± 2.72
	PME5	1 : 5	96.01 ± 3.06	98.57 ± 1.87

^**∗**^Mean ± SD; *n* = 3.
